# EIF5A expression and its role as a potential diagnostic biomarker in hepatocellular carcinoma

**DOI:** 10.7150/jca.58168

**Published:** 2021-06-11

**Authors:** Linjing Li, Xin Li, Qingyan Zhang, Tao Ye, Shuli Zou, Jing Yan

**Affiliations:** 1 Department of Clinical Laboratory Center, The Second Hospital of Lanzhou University, Lanzhou 730000, P.R. China.; 2 Reproductive Medical Center, The First Affiliated Hospital of Sun Yat-sen University, Guangzhou, China.; 3 Guangdong Provincial Key Laboratory of Reproductive Medicine, First Affiliated Hospital of Sun Yat-sen University, Guangzhou, China.; 4 The First School of Clinical Medicine, Southern Medical University, Guangzhou 510515, China.; 5 Department of medicine, Kingsbrook Jewish Medical Center, 585 Schenectady ave, Brooklyn, New York, 11203, USA.

**Keywords:** EIF5A, Hepatocellular carcinoma, Tissue microarray, Diagnostic biomarker, Clinicopathological parameters

## Abstract

**Introduction and objectives:** Eukaryotic translation initiation factor 5A (EIF5A) is a member of the identified eIF family and played an important role in cell proliferation. There are few studies about the correlation between EIF5A and hepatocellular carcinoma (HCC).

**Materials and methods:** We evaluated the expression of the EIF5A in human HCC cell lines and tissues by western blot analysis. Immunohistochemistry analysis of EIF5A was performed on a tissue microarray including 10 normal liver samples and 90 pathological section of HCC. Receiver operating characteristic (ROC) was introduced to obtain an optimal cut-off score for EIF5A positive expression.

**Results:** Western blot results showed that EIF5A was highly expressed in HCC cell lines and tissues. Based on ROC curve analysis, 1/10 (10.0%) of normal hepatic tissues and 67/90 (74.4%) of HCC tissues were tested positive for EIF5A expression, which indicated that EIF5A were significantly up-regulated in HCC tissues compared with normal liver tissues (χ^2^=17.177, *P*<0.001). Furthermore, expression of EIF5A was significantly correlated with histological grade (*P*=0.048), clinical stage (*P*=0.003) and pT stage (*P*=0.003) but not correlated with sex (*P*=0.617) and age (*P*=0.831).

**Conclusions:** In our study, we demonstrated the expression of EIF5A is closely correlated with HCC. In consideration of its relationship with clinicopathological parameters including histological grade, clinical stage and pT stage of HCC, EIF5A could be a potential biomarker.

## Introduction

Liver cancer is one of the most common cancers worldwide and remains the leading cause of cancer deaths in China. In 2018, liver cancer was the fifth most commonly diagnosed cancer and the third most common cause of death in man worldwide 1. Liver cancer is a clinically high incidence tumor and major cause of cancer deaths in China 2. Primary liver cancer mainly includes hepatocellular carcinoma (HCC) and intrahepatic cholangiocarcinoma (iCCA) 3, of which 75%-85% are classified as HCC and 10%-15% are ICC 3,4. Surgical resection remains the preferred treatment for eligible patient. Despite some progress in the mechanism and treatment of the disease, the prognosis of liver cancer remains dismal in the past decades 5. The cure rate of surgical resection for patients with iCCA is 9.7% 6. As we know, the mortality of HCC has been significantly increased in the last 20 years, the burden of economy and medicine will continuously increase 7. However, available clinical methods can only discover HCC patients at relatively late stage 8. Therefore, early diagnosis is urgently required to reduce the mortality of HCC.

Eukaryotic translation initiation factor 5A (EIF5A), an 18-kDa protein, is a member of the identified eIF family 9. EIF5A is involved in initiation and elongation as a translation factor, which is essential for cell proliferation. Importantly, EIF5A contains the special amino acid hypusine (a portmanteau word merging hydroxyputrescine with lysine) which has not been found in any other protein 10. Two isoforms of EIF5A were identified in human respectively, EIF5A1 and EIF5A2. Over expression of EIF5A were found in many malignancies, such as pancreatic cancer 11, cervical carcinoma 12, esophageals quamous cell carcinoma 13 and gastric cancer 14. The relationship of EIF5A and HCC has been studied. EIF5A was found highly abundant in mouse embryonic livers and human HCC cell lines. Using comparative proteomic and genomic approaches and suggested that eIF5A played an important role in HCC tumorigenesis and metastasis 15. However, very few studies explored the correlation between EIF5A and the clinicopathological parameters of HCC. In our study, we found that EIF5A is up-regulated in HCC. It is also closely related with clinicopathological parameters, such as histological grade, clinical stage and pT stage, which might be used as a potential biomarker.

## Material and Methods

### Cell lines and cell culture

Human normal cells LO2 was obtained from laboratory preservation and cultured in Dulbecco's Modified Eagle Medium (DMEM, Gibco, USA) added with 100 units/mL of streptomycin and 100 units/mL of penicillin. Purchased from American Type Culture Collection (ATCC HB-8065, Rockville, MD, USA), HepG2 was cultured in DMEM, containing 10% fetal bovine serum (FBS, Gibco, Life Technologies, Melbourne, Australia). Human hepatoma cell line (Huh7) and Human HCC cell line SMMC-7721 were preserved in laboratory. Huh7 was cultured in DMEM and supplemented with 10% FBS, 10 mM HEPES, 100 units/mL streptomycin and 100 units/mL of penicillin. SMMC-7721 was grown in RMPI 1640(1640, Gibco, USA), which contains 10% FBS, 100 units/mL of streptomycin and 100 units/mL of penicillin. All cell lines were cultured in a 5% CO2-humidified incubator at 37 °C.

### Tissue samples

In this study, a total of 100 paraffin-embedded liver tissue samples were collected from Second affiliated hospital of Lanzhou university from January 2015 to December 2019. Among these paraffin-embedded tissue samples, 10 cases were from patients with normal liver tissues and 90 samples were from patients with HCC without treatment. Clinicopathological information including patients' age at diagnosis, sex, TNM stage, histology grade, clinical stage and pathological diagnosis was collected. The average age of the 100 patients was 50.2 years old. All the details mentioned above are shown in Table 2. This study was performed under the permission of The Ethic Committee of Second affiliated hospital of Lanzhou university and with written informed consent from all patients.

### Western Blot

Cells were washed three times with cold PBS and total proteins were extracted by using Radio-Immunoprecipitation Assay (RIPA) buffer added with 1 mM PMSF (Beyotime, China) in the existence of proteinase inhibitor. Next, the protein concentration was measured by using BCA Protein Assay Kit (Beyotime, China) and a standard BSA curve was used to normalize the values. All the proteins were dissolved in loading buffer (Beyotime, China) and were denaturalized by boiling for 10 minutes. Then, equivalent mixture of each sample was loaded by 10% SDS-PAGE gel electrophoresis and next transferred to polyvinylidene fluoride (PVDF) membranes (Millipore, USA) by a standard wet-transfer equipment (Trans-blot SD, Bio-Rad, USA). The membranes were blocked by using 5% nonfat milk dissolved with Tris-Buffer Saline and 0.1% Tween 20 (1×TBST) at room temperature for two hours. After that, the membranes were incubated with EIF5A primary antibody (Rabbit polyclonal, 1:1000, Proteintech, China) and GAPDH primary antibody (Mouse monoclonal, 1:2000, Proteintech, China), respectively at 4 °C overnight. The membranes were washed for three times with 1×TBST, each for 10 minutes. Afterwards, the membranes were incubated with secondary antibodies labeled with horseradish peroxidase-(HRP) (Goat anti-Rabbit IgG, 1:5000, MultiSciences, China; Goat anti-Mouse IgG, 1:5000, MultiSciences, China) at room temperature for 2 hours. After being washed with 1×TBST again for three times, the membranes were applied with the enhanced chemiluminescence reagents (BeyoECL Plus Kit, Beyotime, China). Protein bands were observed and figures were acquired by Tanon-5200 (Tanon, Shanghai, China). The visual density value of the protein bands was quantitatively analyzed for evaluation.

### Tissue microarrays (TMA) construction and immunohistochemistry (IHC)

The standard method used for the construction of tissue microarrays is described as below. The TMA included 10 normal liver cases and 90 HCC cases. TMA block was cut into sequential tissue sections of 5 μm for immunohistochemical analysis while the staining was performed according to standard procedures. The sections were immersed in xylene and graded ethanol sequentially to be deparaffinized and rehydrated. To inhibit the activity of endogenous hydrogen peroxidase, 0.3% H_2_O_2_ was applied. Then, the sections received antigen retrieval treatment performed by heating with sodium citrate buffer (pH 6.0) for 20 minutes at 100 °C in an autoclave. For the next 30 minutes, the sections were incubated with 1×phosphate-buffered saline (PBS) supplemented with 5% normal goat serum to reduce nonspecific antibody binding. Afterwards, the sections were incubated with EIF5A primary antibody (Rabbit polyclonal, Proteintech, China) at 4 °C overnight. Next day, after being washed with 1×PBS again for three times, the sections were incubated for 30 minutes with HRP conjugated-secondary antibodies at room temperature. Before mounting, the sections were additionally washed with 1×PBS and stained by hematoxylin and got dehydrated. Finally, the sections were visualized by DAB Horseradish Peroxidase Color Development Kit (Beyotime, China).

### IHC Evaluation

With no information of the patients' clinicopathological characteristics, two authors graded independently the immunostaining results of EIF5A. According to the staining intensity and percentage over the entire tumor cells, the two authors applied a semi-quantitative scoring system. Cytoplasmic and membrane staining were interpreted as positive immunostaining in tumor cells. Percentage was calculated by 5% increments (0, 5%, 10%, …100%). Reevaluation of the sections were performed if discrepancy exists.

### Selection of cut-off score

To obtain an optimal cutoff score, receiver operating characteristic (ROC) curve analysis was applied. Meanwhile, the tumor with EIF5A expression was distinguished using 0.1-criterion. Clinicopathological parameters were separated into two groups for ROC analysis by pT stage (T1-T2, T3-T4), clinical stage (I-II, III-IV) and histological grade (G1-G2, G3). Then by plotting the paired specificity and sensitivity of different EIF5A score, the ROC curves were generated. After measuring the distance of each dot to the point [0.0, 1.0] on the curve, the minimum one was selected as the cut-off score. Therefore, if the EIF5A score was below the cut-off score, tumor would be graded as 'negative'. If not, the tumor would be graded as EIF5A 'positive'.

### Statistical analysis

All statistical evaluation was completed with SPSS 20.0 software (SPSS, Chicago, IL, USA). After performed at least three repetitive experiments, measurement data were shown as the mean ± standard deviation (SD). T test was employed to evaluate expression differences within two groups. Receiver operating characteristic (ROC) was introduced to determine the optimal cut-off score for EIF5A immunostaining. Chi-square test was performed to analyze the correlation between the expression of EIF5A and the clinicopathological parameters of HCC. Then the P-value was less than 0.05, the differences are considered statistically significant.

## Results

### Protein expression of EIF5A in HCC cell lines

**Figure 1:**
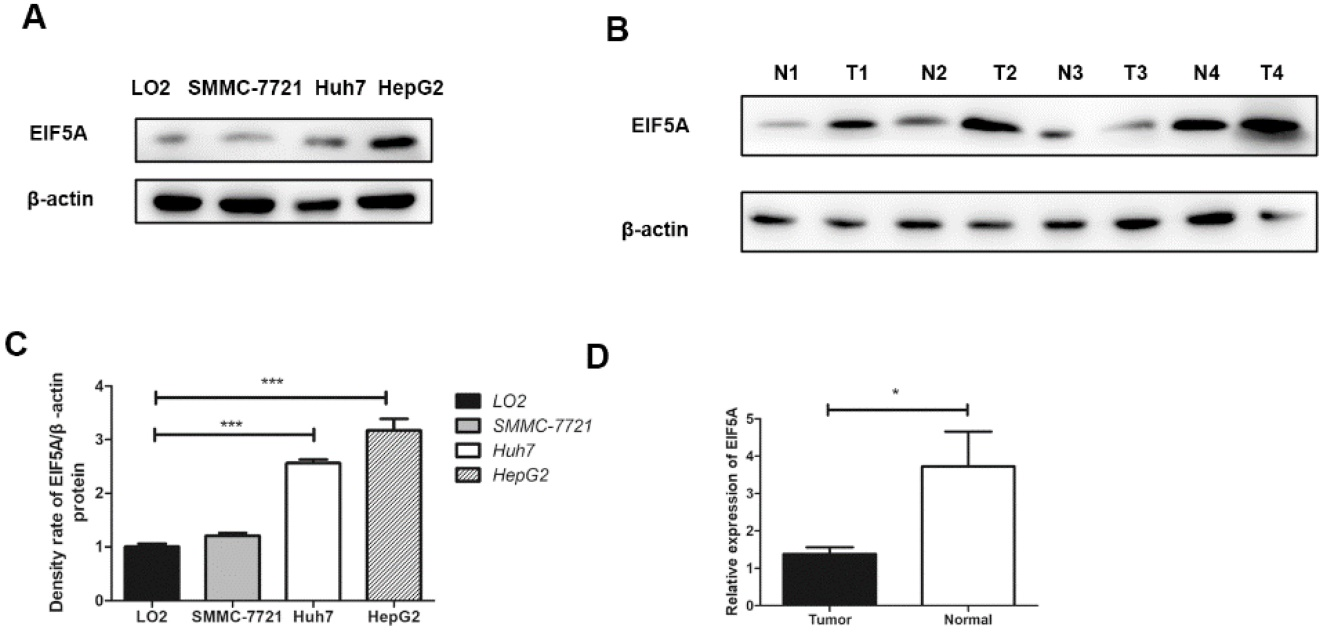
The results of EIF5A expression by Western Blot.** (A,C)** Western blot showed that EIF5A was significantly highly expressed in HCC cell lines (SMMC-7721, Huh7, HepG2) compared normal with hepatic cell line (LO2). **(B,D)** The expression of EIF5A in HCC tissues (T1-T4) were higher than that in hepatic tissues (N1-N4). Western blot results were measured as optical density values and expressed graphically. β-actin was used as internal control (*P<0.05, ***P<0.001).

The expressions of EIF5A in three human HCC cell lines (HepG2, Huh7, SMMC-7721) and LO2 (human normal immortalized cell line) were evaluated by western blot. The results revealed that the expressions of EIF5A in HCC cell lines was significantly higher than normal cell line (Fig. 1).

### Protein expression of EIF5A in tissue samples of HCC

Western blot analysis was used to detect EIF5A expression in 8 paired HCC tissues and normal liver tissues. As we can see in Fig. 1, EIF5A were highly expressed in HCC tissues in comparison with normal liver tissues (*P*<0.05).

### Cut-off score selection and IHC expression of EIF5A in tissue microarray

**Figure 2:**
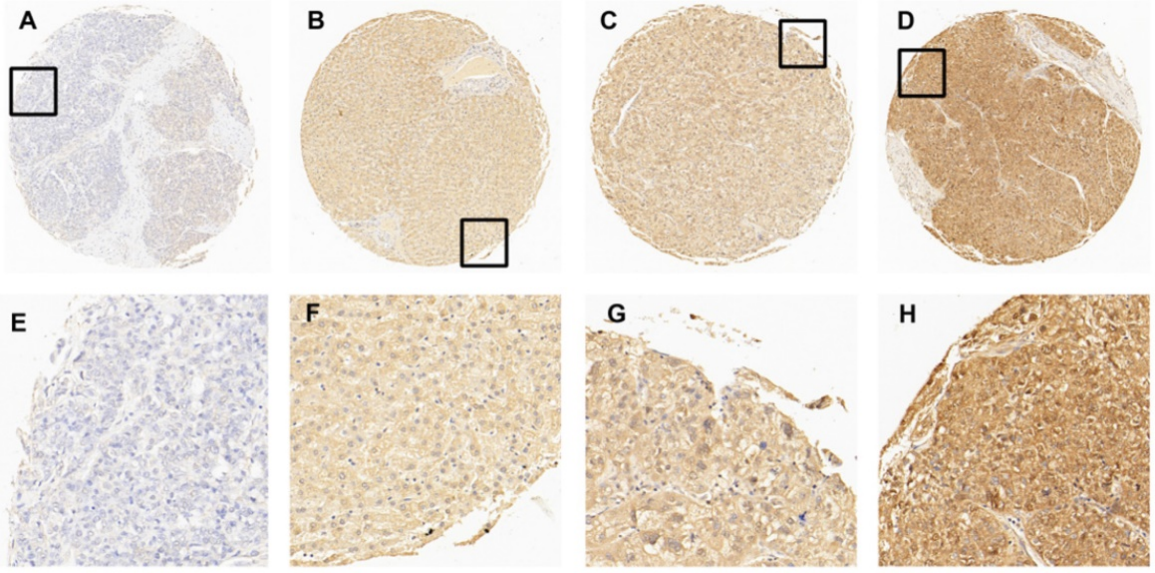
The expression of EIF5A in normal hepatic and HCC tissues by IHC. **(A)** 5% positive expression of EIF5A in normal hepatic tissue (case 117) (10×). **(B)** 40% positive expression of EIF5A in HCC tissue (case 80) (10×). **(C)** 80% positive expression of EIF5A in HCC tissue (case 45) (10×). **(D)** 95% positive expression of EIF5A in HCC (case 61) (10×). The higher magnification (40×) of the part of (A-D) were showed in** (E-H)**, respectively.

In this study, immunohistochemistry (IHC) was performed to evaluate the expression of EIF5A in HCC tissues and normal liver tissues. After detecting the immunoreactivity in the cell membrane of tumor cells, four samples with an increasing expression level of EIF5A were shown in Fig. 2 as representative. Receiver operating characteristic (ROC) curve was employed to select the optimal cut-off score. According to supplementary Fig. 1, among all the clinical parameters, clinical stage and grade and pT stage have the minimum distance from point (0.0, 1.0). Therefore, the cut-off score was 67.5 and above it was defined as EIF5A positive while below it was negative. EIF5A staining was graded as positive in 67/80 (74.4%) and negative in 23/90 (25.6%) among all the 90 HCC tissues according to this cut-off score. Meanwhile, the positive rate in normal liver tissue was 1/10 (10.0%) and negative rate was 9/10 (90.0%). For further analysis, Chi square test was used to explore the correlation of the expression of EIF5A between HCC tissues and normal liver tissues. Again, the results demonstrated that the expression of EIF5A in HCC tissues was significantly higher (Table 1, *P* < 0.001).

### Correlation between protein expression of EIF5A and clinicopathological parameters

Moreover, the relationships between the expression of EIF5A and clinicopathological parameters of HCC were studied. As shown in Table 2, EIF5A expression is significantly correlated with high pT stage, histological grade and clinical stage (all *P*<0.05) but not age or sex (all *P*>0.05).

## Discussion

In 2010, the well-known biomarker α-Fetoprotein (AFP) was not recommended for screen any more by American Association for the Study of Liver Diseases (AASLD) 16. It is revealed that about 30% of early-stage HCC can't be discovered under the use of AFP 17. Since then several serum protein markers have been studied as new biomarkers, such as des-gamma-carboxy prothrombin (DCP) 18, heat shock protein 90 alpha (Hsp90α) 10,20, and Dickkopf-1 (DKK1) 21. Also, miRNA expression was reported to play important roles in carcinogenesis and serves as a prognostic predictor, such as miR-484 22, miR-429 23, miR-590-3p 24. However, the diagnostic accuracy of biomarkers mentioned above is still under investigation. Whether these protein markers should be used alone or in joint use with AFP is unknown.

In our study, we found that EIF5A could be a potential biomarker for earlier diagnosis of HCC as it's closely related with clinical stage. The overexpression of EIF5A has a close connection with tumor, which is readily revealed in many tumors that EIF5A has been considered a possible oncogene 25,26. Increasing evidence have shown that EIF5A participates in many of the known effects of polyamines, such as the viability, migration and proliferation 27. It is revealed that through the sHH signal pathway, EIF5A regulated the proliferation of pancreatic cancer 28. Sonia 29 reported that hypusinated EIF5A directly regulate MYC biosynthesis and promotes growth of colorectal cancer cells. However, overexpression of EIF5A1 was not observed in hepatocellular carcinoma although a correlation between EIF5A1 RNA and liver tumor nodule number was reported 30. For EIF5A2, it has correlation with more advanced stage and/or grade for colorectal, gastric, ovarian and non-small cell lung cancer 31,32,33,34,35 and poor prognosis for bladder, stomach and esophagus cancers 32,33,34,35. But there are little investigations between the connection of EIF5A and the clinical stage of HCC.

To investigate the expression of EIF5A in HCC cells, western blot was performed to compare the differences among three HCC cell lines and one normal liver immortalized human hepatic cell line. HCC cell lines to some extent can represent the biological characteristics of HCC. The expressions of EIF5A were significantly up-regulated in three liver cell lines (HepG2, Huh7, SMMC-7721) compared with the normal liver immortalized human hepatic cell line (LO2) (*P<*0.05).

Furthermore, to evaluate clinicopathological significance of EIF5A, we performed a tissue microarray containing 10 normal hepatic tissues and 90 HCC tissues. Then, a scoring system method based on the percentage of positive staining tumor cells was introduced to evaluate the immunoreactivity of EIF5A. To obtain an optimal cut-off score for IHC evaluation of EIF5A, ROC curve analysis was applied. As shown above, four ROC curves were generated respectively based on different clinicopathological parameters, sex, pT stage, histological grade and clinical stage. Eventually, the optimal cut-off score for EIF5A was set up as 67.5%. Staining above 67.5% was defined as EIF5A positive expression. With this criteria, 1/10 (10.0%) of liver normal tissues and 67/90 (74.4%) of HCC tissues are EIF5A positive. The expression of EIF5A in 90 HCC tissues were significantly up-regulated than those in 10 normal liver tissues (χ^2^=17.177, P*<*0.001). Furthermore, expression of EIF5A was significantly correlated with histological grade (*P*=0.048), clinical stage (*P*=0.003) and pT stage (*P*=0.003) but not correlated with sex (*P*=0.617) and age (*P*=0.831). However, due to the small size of our samples, more *in vivo* and *in vitro* studies are required to further explore the functions of EIF5A in HCC. EIF5A expression is not only a potential marker for HCC diagnosis but also a prognostic marker since it is related to tumor stage.

In conclusion, our study demonstrated that EIF5A was significantly up-regulated in HCC cells and tissues. It is also significantly correlated with tumor grade and stage of HCC, which indicated that EIF5A may be related to the progress of HCC and could be a potential diagnostic biomarker for HCC.

## Supplementary Material

Supplementary figure.

## Figures and Tables

**Table 1:** Relationship of EIF5A expression between normal hepatic tissues and HCC tissues

EIF5A				
	All cases	Negative expression (%)	Positive expression (%)	*P* value
HCC	90	23(25.6%)	67(74.4%)	<0.001
Normal	10	9(90.0%)	1(10.0%)	

**Table 2:** Relationship of EIF5A expression and clinicopathological features in HCC

	EIF5A			
	Negative (%)	Positive (%)	Total	*P* value^b^
**Sex**				
Male	23	53	76	0.617
Female	9	15	24	
**Age (year)**				
≤50.2^a^	18	40	58	0.831
>50.2	14	28	42	
**pT Stage**				
T1-T2	18(39.1%)	28(60.9%)	46	0.003
T3-T4	5(11.4%)	39(88.6%)	44	
**Grade**				
1-2	18(34.0%)	35(66.0%)	53	0.048
3-4	5(13.5%)	32(86.5%)	37	
**Stage**				
I-II	18(39.1%)	28(60.9%)	46	0.003
III-IV	5(11.4%)	39(88.6%)	44	

^a^Mean age; ^b^*P* values are from Chi-square test.
