# Supplementary material for: EIF5A expression and its role as a potential diagnostic biomarker in hepatocellular carcinoma
Source: J Cancer. 2021 Jun 11;12(16):4774–9. doi: 10.7150/jca.58168 (PMC8247388; doi:10.7150/jca.58168)
Supplement: Supplementary file 1 — Supplementary figure. [file jcav12p4774s1.pdf]

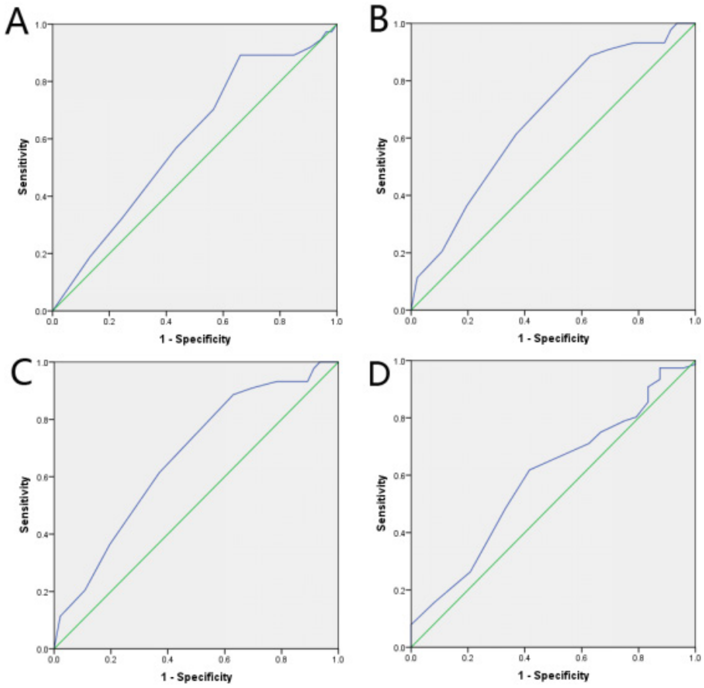

Supplementary Figure 1. The optimal cut-off score(67.5%) for positive and negative expression of EIF5A measured by Receiver Operating Characteristic (ROC) and blue line is as ROC curve： A. pT stage. B. Grade. C. Clinical stage. D. Sex.
